# Supplementary material for: Are carbon emissions trading and green financial instruments synergistic? -Comprehensive quantitative research based on content analysis
Source: PLoS One. 2024 Mar 7;19(3):e0298601. doi: 10.1371/journal.pone.0298601 (PMC10919601; doi:10.1371/journal.pone.0298601)
Supplement: S1 Data — (ZIP) [file pone.0298601.s001.zip › Data/地级市Z_F工作报告.zip!/╔┬╬≈/║║╓╨╩╨/└┤╫╘╣1⁄2╓┌║┼í╛┬φ┐╦╩2╛▌═°í┐_2018.docx]

各位代表：

　　现在，我代表市人民政府向大会作工作报告，请予审议，并请市政协委员和其他列席人员提出意见。

　　一、2017年工作回顾

　　过去一年，是本届政府工作的开局之年，是汉中发展取得丰硕成果的一年。一年来，全市上下认真学习领会习近平新时代中国特色社会主义思想，突出全面迎接、贯彻落实党的十九大这条主线，在省委、省政府和市委的坚强领导下，紧扣追赶超越定位、“五个扎实”要求和“五新”战略任务，聚焦“三市”目标，统筹推进稳增长、促改革、调结构、惠民生、防风险各项工作，经济社会发展保持平稳向好态势，圆满完成了市五届人大一次会议确定的各项目标任务。

　　——追赶超越取得新成效。实现生产总值1333.3亿元，增长9.6%，增速居全省第2位、创近五年来最好成绩。地方财政收入45.6亿元，同口径增长9.6%；财政支出311.7亿元，增长11.3%、增速居全省第4位。城乡常住居民人均可支配收入分别达27812元、9666元，增长8.7%、9.2%，增速居全省第1、第3位。

　　——发展动能实现新突破。西成高铁、宝汉高速汉中境内建成通车；略钢全面复产，公主牌香烟恢复上市并取得全国销售权，陕飞扩能改造主体建成投用；市投资控股集团组建运行；各类市场主体增长22.7%，新培育“四上”企业469户。

　　——城市品位有了新提升。兴汉生态文化旅游园区核心区即将建成，一江两岸湿地公园和天汉文化公园C、D区开园投运，“国卫”复审顺利达标，“国园”创建一举成功。

　　——真美汉中再添新名片。“汉中仙毫”跻身“中国优秀茶叶区域公用品牌”，“汉中三堰”入选世界灌溉工程遗产名录，我市荣获“全国幸福家庭示范市”称号。

　　——创新实践呈现新亮点。健康扶贫“汉中模式”全国推广；我市成为“中国最美乡村旅游目的地”，全域旅游工作经验在全国会议上交流；城固机场年旅客吞吐量达41.4万人次，增长115%、在西部机场集团通航机场中居第1位。

　　一年来，我们主要做了以下工作：

　　（一）全面出击精准发力，扎实推进脱贫攻坚。坚持精准扶贫精准脱贫方略，围绕“两不愁、三保障”，全面落实“八个一批”帮扶举措，可望顺利实现5.72万人脱贫、105个村退出和佛坪县摘帽的年度目标。整合财政涉农资金21.6亿元，投入财政专项扶贫资金2.6亿元；建立30亿元扶贫基金和10亿元农业发展专项基金，探索推行“投贷联动”新模式，覆盖5个县14个镇28个贫困村。以“三变”改革为抓手，强力推进产业扶贫，建成扶贫互助和土地股份合作社1170个，带动1.9万户5.8万贫困人口稳定增收。534处农村安全饮水工程、42个村通村路、270个村标准化卫生室建设和124个村农网升级改造全面完成，易地搬迁9046户、新开工住房19737户，农村危房改造12866户。“三大帮扶体系”全面落地，汉中与南通扶贫协作深入推进，实施项目117个、完成投资1.23亿元，带动3.5万人增收。全省教育扶贫现场会在汉中召开，我市公益性岗位开发安置贫困户、土地政策助力脱贫和留坝县村级扶贫互助合作社、勉县土地股份合作社等经验做法受到中省肯定。

　　（二）坚持不懈转型升级，力促产业提质增效。持续强工稳农兴三产，推动产业发展向中高端特色化迈进。工业经济回升向好。出台支持工业稳增长23条措施，规上工业总产值1424.88亿元、增长23.5%，增加值440.75亿元、增长13.7%、增速居全省第4位；21个省级工业园区实现营业收入980亿元、增长13%。农村经济稳步发展。全面推进小农户对接大市场，加快发展现代农业，第一产业实现增加值210亿元，增长5.1%、增速居全省第1位。粮油生产保持平稳，培育壮大特色产业和新型经营主体成效明显。洋县成功创建全国朱鹮生态保护产业知名品牌示范区，全市地理标志商标达18件、居全省第1位。旅游三产持续繁荣。汉山、金沙湖景区建成开园，熊猫谷成功创建4A景区，实现旅游总收入232亿元、增长34%。商贸物流、金融保险、健康医疗、养生养老等现代服务业蓬勃发展，社会消费品零售总额421.05亿元、增长14.1%、增速居全省第3位。

　　（三）全力以赴扩大投资，强化项目拉动作用。把项目建设作为促投资稳增长的有力抓手，坚持领导包抓促动、挂图作战推动、集中开工牵动、现场观摩带动、内引外联驱动，项目建设取得突破性进展。全社会固定资产投资1443.21亿元，增长19.3%、增速居全省第4位；304个市级重点项目实现投资712.5亿元，完成计划的108.8%。汉江综治左岸堤防建设全面收尾，引汉济渭、阳安铁路扩能改造、火车站南北广场改扩建、酒泉至湖南800千伏输电线路等项目进展顺利，西镇高速、天汉大剧院等项目开工建设。

　　（四）持之以恒统筹城乡，加快打造最美城市。大力推动城乡统筹和区域协调发展，陕西最美城市建设迈出新步伐。中心城区城市建设重点年活动卓有成效，431个城建重点项目完成投资405亿元；打通了西环路南段、大河坎东昌路等一批“断头路”，汉宁路地下综合管廊等项目进展顺利，博望二路、龙亭北路等工程开工建设，东关、南关片区改造有序推进。深入实施“五城联创”，大力开展城市环境综合整治“百日会战”和西成高铁沿线环境整治，县城、重点镇和美丽乡村建设扎实推进。留坝县荣获国家卫生县城称号，镇巴、宁强县成功创建省级森林城市，佛坪县跻身中国美丽乡村建设示范县，青木川镇入选全国美丽宜居示范小镇，武侯镇列入第二批全国特色小镇，建成市级美丽乡村旅游精品村20个。

　　（五）标本兼治优化生态，大力改善环境质量。强力推进“铁腕治霾·保卫蓝天”雷霆行动，中心城区空气质量优良天数269天，重污染天气较上年减少4天。大力实施水污染防治五年行动计划，完成中心城区及8个县城污水处理厂提标改造并实现A标排放，推行“河长制”走在全省前列，汉江、嘉陵江流域水质保持优良。严格落实“土十条”，初步建立土壤环境质量监测防控体系。节能减排任务全面完成。造林23.77万亩、其中退耕还林3.5万亩，治理水土流失850平方公里，建成高标准农田13.2万亩，永久基本农田划定工作获国土资源部、农业部通报表扬，成功创建省级环保模范城市，留坝被命名为全国首批“两山论”实践创新基地。中央环保督察组和西北督查中心反馈问题整改扎实有效，累计问责555人次。

　　（六）改革完善体制机制，释放创新发展活力。钢铁、煤炭去产能后续工作和“地条钢”专项整治扎实有效；商品房库存消化周期下降为9.3个月；市投资控股集团设立1支母基金和10支子基金，支持扶贫、小微企业发展等领域投入6.93亿元；落实营改增、直供电等政策，为纳税户减免税费25亿元。市级行政审批事项精简率64.8%。汉中经济开发区、旅游发展、城市综合执法、盐务管理等方面体制改革任务顺利完成，南郑撤县设区。国企“三供一业”分离移交协议全部签订，略钢和市药材总公司、粮油总公司、收获机械厂改制基本完成。农村集体产权制度改革试点和国有林场改革全面完成。国家创新型试点城市加快建设，市绿色循环暨产业经济研究院组建运行；申报发明专利730件，培育发展“民参军”企业30余户，新发展高新技术企业15家，技术合同交易额1.8亿元，科技进步贡献率43.6%。深入实施“四向融入”战略，出台鼓励“汉商”回归创业十条优惠政策及细则，积极赴京津冀、长三角、粤港澳等区域开展招商活动，新城投控、绿地集团、众合创投、西安迈科、红星美凯龙等一批大企业来汉投资。全年引资到位620亿元、增长20%，其中省际资金571亿元、增长36%；进出口贸易总额9.1亿元，增长30%。

　　（七）不遗余力改善民生，持续增进群众福祉。民生类支出258.5亿元，占总支出的83%。城镇新增就业5.9万人、登记失业率3.2%，转移农村富余劳动力82.28万人；在全省率先实行城乡居民医保市级统筹和城镇基本医保异地结算，企业退休人员基本养老金实现“十三连涨”。城乡低保保障6.2万户12万人，五保供养1.99万人，医疗救助、临时救助18.32万人次。提前一年完成国家义务教育均衡县和省“双高双普”县创建任务，高考实现“六连升”。深化医改取得新成效，市级公立医院全面取消药品加成并同步调整医疗服务价格，组建全省首家健康管理联合体。新建和改扩建各类养老机构132个，新增养老床位3470张，每千名老人拥有床位达到37张。全年实施棚户区改造15939套，累计分配公租房3.7万套。住房公积金支持住房消费资金26.47亿元，资金使用率居全省第2位。市群艺馆建成投用，创建省级文化先进县3个，216个“两馆一站”全部免费开放，组织文化下乡、惠民演出、公益电影放映等2.53万场次，纪录片《汉中天坑》《味道》在央视播出，广播剧《汉中秘密特使》荣获省“五个一工程”优秀作品奖，《风从巴山来》荣获省第八届艺术节“文华剧目奖”，镇巴民歌登上央视、走出国门，汉中书画作品展首次在京举办。市第六届运动会圆满成功。荣获全国第三次农业普查工作先进单位。社会治理、安全生产、食品药品安全监管和防灾减灾扎实有效，社会大局和谐稳定。

　　（八）久久为功从严治政，全面提升政府效能。扎实推进“激情、干净、超越”主题作风建设和“思想奠基·党性塑造”工程，严格落实“两个责任”和“三项机制”，驰而不息转作风、提效能。坚持市政府班子集体学法和重大事项专家咨询制度，严格执行政府性债务、产业资金、国有资产（配置）等管理办法，决策民主化、科学化水平不断提升。自觉接受人大、政协监督，全年邀请省、市人大代表、政协委员120余人次列席市政府重要会议，人大代表建议、政协提案办复率100%、满意率98%以上。办理“市长信箱”来信、人民网留言2771件，回复率100%；市级“12345”政府服务热线平台建成投运；市、县区行政服务中心受理审批事项193.1万余件、办结率99.9%；公布了行政事业性收费、政府性基金和市级部门行政许可中介服务收费目录清单。贯彻八项规定精神长效机制进一步健全，全市“三公”经费下降3.3%。在年度目标责任考核满意度调查中，“两代表一委员”满意度居全省第1位，人民群众满意度、营商环境满意度、作风建设满意度均居全省第3位。

　　与此同时，审计监督进一步加强，人防体系平战结合能力明显提升，质量强市战略深入推进，物价水平总体平稳，民族宗教领域保持和谐，供销社改革持续深化，外事侨务工作全面加强，档案、地方志服务发展水平不断提高，老龄、慈善、残疾人、红十字会事业较快发展，民兵和预备役等工作也取得了新成绩。

　　各位代表，一年来，我们始终保持战略定力、勇敢面对挑战，千方百计稳增长、提质量，壮大发展新实力；我们牢牢把握时代大势、创新思路举措，综合施策补短板、强弱项，培育发展新动能；我们时刻牢记历史使命、激情干净超越，持续发力务实功、求实效，推动发展新跨越。放眼天汉大地，春潮涌动、活力迸发，汉中已站在新起点、步入新时代。这是省委、省政府和市委坚强领导的结果，是市人大、市政协监督支持的结果，是全市上下团结奋斗的结果。在此，我谨代表市人民政府，向全市广大干部群众，向驻汉人民解放军、武警官兵，向大力支持政府工作的各位代表、各位委员、各民主党派、各人民团体和社会各界人士，表示衷心的感谢并致以崇高的敬意！

　　回顾过去一年工作，我们也清醒地看到汉中经济社会发展中存在着一些矛盾和问题：一是项目储备不够，缺少带动性强的大项目、好项目，抓机遇的意识不强，项目支撑发展后劲不足。二是受市场、成本及产业链等因素影响，钢铁、有色行业效益空间有限，工业稳增长动能不强。三是财政增收空间较小，盘活存量和优化支出尚不到位，收支矛盾依然突出。四是贫困人口多、群众收入低，如期脱贫摘帽、实现全面小康的任务十分艰巨。五是法治政府建设和作风建设有待深化。对此，我们一定坚持问题导向，强化责任意识，全力攻坚克难，认真加以解决。

　　二、2018年工作目标及思路

　　今年是全面贯彻党的十九大精神的开局之年，也是汉中实施“十三五”规划和追赶超越建设“三市”必须取得重大突破的关键之年。做好今年工作，我们必须站位全局，综合研判，明晰方位，在新时代追赶超越建设“三市”中赢得主动、赢得优势、赢得未来。

　　从发展机遇看，在党的十九大确立的新思想、新战略引领下，推进“三大变革”、深化供给侧结构性改革、构建现代化经济体系、实施乡村振兴等战略举措，为我们对接发展优势、补齐短板弱项提供了有力支撑；国家推进西部大开发形成新格局、实施苏陕和津陕合作、支持革命老区和深度贫困地区加快发展，省委、省政府推进新时代追赶超越、实施“五新”战略和推动陕南融入汉江生态经济带、支持汉中建设生态文化旅游名市的决策部署，为我们进一步争取支持、加快发展提供了有利环境；“一带一路”倡议得到普遍响应、长江经济带建设加快实施和陕西大力发展“三个经济”，为我们推进融合发展、建设丝路源点城市创造了广阔空间。从面临挑战看，我市经济总量不大、产业结构不优、发展要素不全、创新能力不强，破解发展不平衡不充分问题任重道远；省内和周边城市竞相发展势头逼人，前甩后追的压力依然很大；“十三五”进入第三年，“三市”建设处于攻坚阶段，实现发展大突破的任务艰巨而紧迫；西成高铁开通是机遇、更是挑战，防止虹吸效应的课题严峻地摆在我们面前。从未来潜能看，随着“六大产业集群”发展壮大，航空智慧新城、兴汉新区、滨江新区加速崛起，“三大项目支撑体系”持续发力，一批新的优势产能和增长板块不断涌现，必将为汉中新时代追赶超越建设“三市”注入强劲动力。总体来讲，2018年是汉中各项工作大攻坚、大突破之年。我们要全面把握机遇，沉着应对挑战，推进发展行稳致远。

　　全市经济社会发展的总体要求是：坚持以习近平新时代中国特色社会主义思想和党的十九大精神为指引，深入贯彻中央经济工作会、省委十三届二次全会和市委五届五次全会精神，坚持新发展理念和稳中求进总基调，统筹推进“五位一体”总体布局、协调推进“四个全面”战略布局，牢牢把握追赶超越和“五个扎实”要求，聚焦“五新”战略任务，突出绿色循环、生态宜居发展定位，坚持高质量发展与中高速增长有机统一，以供给侧结构性改革为主线，推动“三大变革”，发展“三个经济”，打好“三场攻坚战”，全力以赴稳增长、促改革、调结构、惠民生、防风险，奋力谱写汉中新时代追赶超越建设“三市”新篇章。

　　主要预期目标是：生产总值增长10%左右，全社会固定资产投资增长20%左右，规模以上工业增加值增长12%以上，社会消费品零售总额增长14%，财政总收入增长10%，地方财政收入增长8%，城乡常住居民人均可支配收入分别增长8.5%、9.2%。实现上述目标，我们将从四个方面着力：

　　第一，贯彻新要求，坚持规划引领、项目支撑推进发展。紧紧围绕转方式、调结构、促升级，加快建设现代化经济体系，将高质量发展要求落到实处。要坚持目标导向、强化规划拉动作用，加强各类规划实施情况的研判评估，对发展目标进行修正、提标，对发展路径进行完善、优化，确保各项工作始终在规划引领之下有效推进；要坚持项目支撑、擎起发展“四梁八柱”，围绕做强做大存量、积极引入增量，及时谋划和跟进项目，使规划从线路图变为结构图再到施工图，突破正负零，真正做到以项目支撑发展；要坚持以终为始、全力以赴追赶超越，咬定发展目标，高起点谋划、高标准推进，实现后来居上。

　　第二，紧扣新变化，坚持抓住重点、突破难点力促落实。我国社会主要矛盾的新变化，是我们做好各项工作的着力点和突破口。要抓重点、以点带面，聚焦事关改革发展全局的关键领域和重要环节，分清轻重缓急，精准发力、重点突破、带动全局；要破难题、抢占先机，针对脱贫攻坚、乡村振兴、环境保护、县域发展、民营经济等方面具体问题，对内挖掘潜能、对上争取支持、对外吸纳要素，补齐短板、强化弱项；要重落实、善作善成，建立健全横向到边、纵向到底的责任体系，全力以赴抓好落实，不达目的决不罢休。

　　第三，站位新格局，坚持改革发力、开放借力追赶超越。全面深化改革和全方位开放的新格局，为我们释放发展活力创造了良好条件。要向改革要动力，发挥市场资源配置的决定性作用和更好发挥政府作用，推动“三大变革”，充分激发企业、县域、市场、人才等方面的活力；要用开放聚要素，坚定不移实施“四向融入”战略，打造开放新高地，全力吸纳外部要素，使其与汉中资源发生“化学反应”，推动发展实现从量到质的飞跃；要靠融合补短板，以更宽的视野审视外部资源，以全新的思维谋划融合发展，善取他山之石、善于合作借力，最大限度释放动力潜能，努力实现经济总量追赶、质量效益超越、发展模式创新。

　　第四，聚焦新使命，坚持顺势有为、激情担当建设“三市”。主动顺应汉中步入新时代的发展大势，不忘初心使命，勇于担当作为。要知行合一、止于至善，学深悟透中省决策部署，培养专业能力、弘扬专业精神，真正做到发展实践与中省部署高度统一、发展优势与良好机遇充分对接；要焕发激情、苦干实干，用好“三项机制”，完善绩效管理和考核评价体系，用考核倒逼责任落实，以奖惩激发工作活力，在阳光下晒成绩、为担当者站好台；要同心同向、聚力前行，齐心协力抓发展，全力推动汉中新时代追赶超越建设“三市”实现新跨越。

　　三、今年工作的重点任务

　　实现新时代追赶超越建设“三市”新突破，我们将围绕打好“十场重点战役”、聚焦补齐“十大短板弱项”，切实抓好八个方面工作：

　　（一）强推重点项目建设，做强追赶超越建设“三市”的战略支撑。坚定不移地实施项目拉动战略，以项目大突破推动汉中大发展。全年实施重点项目458个，年度投资853.8亿元，带动固定资产投资1740亿元。

　　高水平策划包装。按照高质量发展与中高速增长有机统一的要求，依托资源禀赋和产业布局，聚焦“三大项目支撑体系”和“六大产业集群”，树立大产业、大园区、大基地理念，着眼高端装备制造、新型能源利用、新兴产业发展、生态旅游开发和基础设施完善，谋划一批大项目、好项目，特别是一批投资百亿元左右的支柱型项目和延链补链强链的产业类项目，确保项目接替有序、支撑有力。

　　大力度推进建设。继续推行领导包抓、观摩点评、挂牌督办等措施，实施重点项目计划动态管理、投资增速和进入市级项目盘子加扣分考核，倒逼重点项目加快建设，确保华阳旅游专线等项目建成投用，阳安铁路扩能改造、西镇高速、244国道汉台至南郑、黎坪旅游专线、引汉济渭等项目加快推进，108国道洋县过境段、317省道洋县至勉县段、镇巴星子山隧道及引线工程、秦巴深山环线部分规划路段和青海至河南800千伏输电工程汉中段等项目开工建设，力促汉中机场扩建、汉巴渝快速铁路、兰渝铁路阳平关连接线等项目取得突破性进展。

　　全方位招大引强。用好丝路源点城市和“张骞故里文化经贸使团”名片，全面落实“汉商”回归政策，瞄准京津冀、江浙沪和关天、成渝、江汉经济圈等重点区域，紧盯国际国内500强、行业100强等重点企业，主动走出去深度对接，真诚请进来务实合作，着力引进一批大企业、大集团。全年引资到位突破700亿元、增长14%。

　　优质化服务保障。强化主动服务、高效服务理念，着力打造立项审批、跟踪落实、衔接联系、现场办公的服务链条，构建无缝对接、畅通无阻的绿色通道。深化政银企合作，帮助县区建立投融资平台，全力激活民间资本，不断拓宽融资渠道，确保每个重点项目都有2至3套融资备选方案。狠抓土地资源优化配置和集约利用，建立由政府主导的大收储制度和土地收储、规划、开发的联动机制，规范土地储备融资行为，增强政府对建设用地的调控和保障能力；按照国土资源部相关办法，严格处置闲置土地；用好占补平衡、增减挂钩政策，全力保障项目用地。加强建设环境整治，严厉打击蓄意敲诈、恶意阻工、强揽工程等行为。

　　（二）完善现代工业体系，培育追赶超越建设“三市”的强劲动能。坚持质量第一、效益优先，全面落实中国制造2025汉中实施意见，构建富有竞争力的现代工业体系。

　　做强装备制造。大力实施军民融合战略，依托陕飞公司等企业集团进行产业布局，推动航空零部件加工配套协作、转包生产和辅业转移，加快实施民用货机项目，力争建成全国军民融合示范基地；抓好万目仪电扩产迁建、汉江工具新一代切削工具、燕航精工AMT电控节能变速箱生产线、汉机精密螺杆转子产业化等项目，提高协作配套率，不断做强装备制造业。

　　提升现代材料。协调推进陕钢汉中公司和汉钢公司产业协作，发展短流程冶炼，延伸产业链条，加快转型升级。全力推进洋县现代材料产业园建设，抓好盛华公司铁钒分离、北京首铝铝模板生产线等项目。鼓励支持石墨烯、硅石等新材料产业开发，促进现代材料产业延长链条、提升水平。

　　扩张高品质食药。持续抓好公主牌香烟扩销促产，确保年销量超过1万大箱；支持城固酒业、秦洋长生酒业等地方酒企做大做强；引进大企业大品牌，开发高端饮用水、饮料产品和食用油；加快天然谷医药产业园、汉王药业现代中药产业园、汉江药业高端原料药产业园建设，不断提升产品附加值和市场竞争力。

　　培育新兴产业。积极适应智能化大趋势，开发运用大数据平台，加快培育新一代信息技术、智能制造、人工智能、无人机制造应用、工业机器人等战略新兴产业。坚持做大整车、做强配套、做优服务，打造国内领先的新能源、智能网联汽车产业集群，力争2021年全市各类汽车产能达到10万辆。今年重点抓好八度阳光黑硅制绒与电池组件封装生产线、金汉新能源汽车全系列产品生产基地、君毅无人机总装等项目，扩大新兴产业贡献份额。

　　开发新型能源。加大衔接力度，推动镇巴石油天然气勘探开发、LNG分布式能源项目、宁强至西乡天然气管道建设取得实质性进展，加快天然气热电联产等项目建设。谋划实施光伏、氢能、生物质能等新能源开发利用项目，着力培育一批朝阳产业和新的增长点。

　　壮大市场主体。全面落实减税降费等政策，从投资融资、奖励激励等方面，支持企业向集群化、集团化发展。积极构建亲清新型政商关系，激发和保护企业家精神，弘扬劳模精神和工匠精神，壮大企业家队伍，激发民营企业活力。做好第四次全国经济普查，加快培育“四上”企业，实施中小企业成长梯队培育工程，认定一批“专精特新”中小企业，全年新增“四上”企业360户、中小微企业6300户、个体工商户1.6万户。全面落实工业稳增长政策措施，全力保障生产要素，力促工业提质增效，全年完成规上工业总产值1550亿元、增长15%。

　　（三）实施乡村振兴战略，筑牢追赶超越建设“三市”的坚实基础。认真贯彻落实中央1号文件和中省农村工作会议精神，大力实施乡村振兴战略，推动实现农业强、农村美、农民富。

　　打好脱贫攻坚战。围绕“两不愁、三保障”，注重从打赢向打好转变，坚持扶贫扶志扶智相结合，精准严实推进脱贫攻坚，确保461个村退出、18万人脱贫和汉台区整体退出、留坝县脱贫摘帽。加大项目建设和涉农资金整合力度，加快水、电、路等基础设施和公共服务配套建设。深化金融扶贫，着力破解产业发展、基础设施建设资金瓶颈。抓好产业扶贫，引进一批社区工厂，发展壮大特色产业，着力构建增收脱贫长效机制。巩固提高健康扶贫成果，精准落实易地移民搬迁、危房改造、就业、教育、生态、兜底保障等扶贫措施。深化“三大帮扶”行动，建立大扶贫格局。推动苏陕扶贫协作，抓好苏陕产业园中园建设，引导南通企业来汉设立分支机构和扶贫车间。落实各项政策举措，全力支持镇巴、略阳2个深度贫困县和110个深度贫困村加快脱贫步伐。

　　发展特色现代农业。深化农业供给侧改革，持续推进十大绿色农产品基地提档升级，加快推动粮油、蔬菜、水果、茶叶、畜牧等农产品精深加工。积极发展林下经济、休闲农业、创意农业等新产业新业态，着力抓好洋县龙亭、宁强氐羌、西乡五丰等田园综合体开发建设，规划打造一批农业公园，促进一二三产融合发展。大力推进品牌强农，抓好“三品一标”认证管理，有效整合特色品牌，进一步提升“汉中仙毫”等“汉字号”农产品在全国的影响力。

　　推进小农户对接大市场。积极培育龙头企业、农民合作社、家庭农场、职业农民等新型经营主体，支持龙头企业进入资本市场，创新经营主体与农户之间的利益联结机制；大力发展订单农业和智慧农业，鼓励开发高端农产品；在京津沪浙等一线城市设立汉中名优产品专营店、展销厅，举办系列推介促销活动，打通产加销环节，持续推动农业增效、农民增收。

　　建好美丽宜居乡村。做好乡村规划编制，行政村规划编制覆盖率达到80%以上。全面建设“四好农村路”，实施农村通村通组沥青（水泥）路、安全饮水巩固提升和自然村通动力电等基础设施“三提升”工程；大力开展农村人居环境整治三年行动计划，推进农业面源污染治理和村容村貌整治，抓好10条美丽乡村风景线和105个市级示范村建设。组织做好第10次村委会换届工作。健全自治、法治、德治相结合的乡村治理体系，不断提高乡村社会文明程度。

　　稳步推进农村改革。健全城乡融合发展体制机制，消除要素下乡各种障碍。持续推进土地制度改革，有序流转农村土地，大力发展多种形式的适度规模经营，探索宅基地所有权、资格权、使用权“三权分置”。抓好农村集体产权制度改革，推进“三变”改革和县级农村产权交易中心建设，壮大农村集体经济。开展农业大灾保险试点，鼓励引导工商资本参与乡村振兴。探索设立由市县政府出资、涉农企业参与合作的乡村振兴服务公司，以政府购买服务等多种方式，破解农村在项目建设、产业发展、资金使用、人才培训等方面的难题。

　　（四）提升旅游三产水平，开拓追赶超越建设“三市”的广阔空间。抢抓高铁开通的黄金机遇，推动旅游三产大繁荣。全年接待游客突破5000万人次、增长18%以上，旅游总收入超过300亿元、增长25%以上；社会消费品零售总额超过480亿元。

　　深化全域旅游。围绕打造“一老家四基地”，以创建国家全域旅游示范市为抓手，力促兴汉新区汉文化旅游示范区“五一”开园、紫柏山创5A通过国家旅游局资源评估，加快华阳、黎坪、青木川等景区提升，抓好龙岗文化生态旅游园、两汉三国文化景区、天汉文化公园、古路坝研学小镇、南湖和红寺湖旅游综合开发、羌文化博览园等项目建设，推进九昱温泉、丝路花海等休闲度假、乡村旅游项目开发。实施“旅游+”战略，促进旅游与文化、生态、农业、体育等全面融合，积极培育新产品、新业态。发展100家农家宾馆、农家乐、民宿酒店和一批旅游商业街、美食街；围绕居家生活、休闲娱乐、商务会议等定位，结合实施乡村振兴战略，精心培育打造“汉家乐”品牌。从业态建立、税收优惠、平台搭建、设施配套、社会服务和住房政策衔接等方面入手，规划建设一批“产权式”酒店、“酒店式”公寓，给旅居汉中人士提供方便。深入研究、加强衔接，吸纳西安、成都等区域最具特色的旅游要素进入汉中。抓好旅游集散中心建设，严厉整治旅游环境，创新油菜花节等品牌活动，优化旅游线路，提升服务水平和全民参与度，扩大汉中旅游影响力。

　　优化三产服务。深入推进“互联网+”行动，统筹解决物流、场地、设备、人才、合作方式等问题，大力发展农村电子商务，实现所有行政村电商全覆盖；推动传统商贸线上线下融合发展，新培育电商企业50户，建成1个省级示范企业、12个市级示范企业。加快发展连锁经营、仓储经营、融资租赁，大力培育现代物流、现代金融、养生养老、康体健身等产业，鼓励发展数字经济、共享经济。抓好汉府长乐坊、华汉新世纪、普汇中金世界港等30个商贸流通项目，加快公益性农产品批发市场建设，改造提升一批农贸市场，不断完善城乡市场流通体系。

　　扩大城乡消费。深入开展放心消费创建和十大消费行动，充分发挥地理标志产品和老字号品牌引领作用，做精做实扩需促销活动。加大住房公积金归集力度，促进住房消费。大力培育汽车、医养结合等消费热点，积极推动服务消费、信息消费、品质消费、时尚消费，更好满足城乡群众消费需求。

　　（五）促进区域城乡协调，打造追赶超越建设“三市”的良好平台。加快构建以中心城市为核心、县级城市为节点、特色名镇为支撑的新型城镇体系，推进区域城乡协调发展。

　　强化中心城区带动。完成新一轮城市总规修编，实施中心城区美丽城市建设三年计划，推动“五个十大”重点工程，加快打造空间优、交通畅、功能全、生态美、特色显的美丽城市。全年实施重点项目107个，年度计划投资323亿元。全力推进沿江排污口综合治理、南环路雨污分流改造，年内解决雨污混流问题；抓好兴汉新区跨铁路道路、汉宁路地下综合管廊、汉中综合客运枢纽站等重点项目，打通望江路、南一环西段、龙岗大道、天汉大道南段等“断头路”，加快两座跨江大桥、出城快速干道、城市轨道交通等项目前期工作；实施南关片区等区域棚改1.2万户。同时，着眼长远，统筹推动各大板块协调发展，加快老城区改造提升，注重历史文化遗址保护开发，做强文化底蕴；突出兴汉新区示范引领，全力打造中国汉文化传播示范基地，提高城市品位；推动滨江新区加速崛起，完善公共服务设施，打造科技创新基地，建设高品质宜居新城。

　　巩固城市创建成果。加大城市综合执法力度，更好发挥城市管理执法与街道社区单位协同作战作用。强化物业收费及供水、供电、供气、停车等服务质量监管，提升城市公共交通服务质量和保障能力。大力推进城市修补、生态修复和卫生保洁、增绿补绿、市容美化、环境整治，规划建设一批公厕、停车场、充电桩、人行天桥等公共设施，持续抓好市民素质提高、文明素养养成，巩固国卫、国园和省级环保模范城市、省级文明城市创建成果，加快国家森林城市、省级生态文明建设示范市创建步伐，启动国家食品安全示范城市和节水型城市创建工作。

　　增强城镇承载能力。立足区位、资源、人文、产业特点，启动县城总体规划修编工作。围绕高标准建设示范县城，加快完善公共设施，提升绿化美化水平，增强承载能力，完成重点项目投资20亿元以上。坚持项目支撑、产业带动、文化引领、生态提升，加快25个省市级重点镇、文化旅游名镇建设，完成年度投资62亿元，发挥好建制镇承接县城辐射、带动乡村振兴的重要作用。

　　推动县域经济升级。出台支持县域经济发展的政策措施，设立县域经济发展基金，对接市县资源、联动招商引资、强化苏陕合作，支持各县区发挥优势、错位发展，板块开发、整体联动，促进汉台、南郑加快强区步伐，城固跻身全省十强县，洋县、勉县、西乡争先进位，宁强、镇巴、略阳、开发区转型升级，留坝、佛坪旅游兴县。落实好促进园区创新发展的实施意见，围绕发展枢纽经济、门户经济、流动经济，构筑区域创新高地、产业高地、开放高地，立足把产业园区建设成体制机制创新先行区、创新驱动集聚区、绿色循环产业引领区、高水平营商环境示范区，加快建设汉中国家级经济技术开发区、汉中高新技术产业开发区、兴汉新区汉文化国家生态旅游示范区、航空智慧新城国家级军民融合航空产业及物流示范区，着力打造8个产值过百亿元的县级园区，形成全市产业集成、集约、集群发展新格局。

　　（六）改革开放创新驱动，激发追赶超越建设“三市”的生机活力。抓住重点领域，深层次推进改革，全方位扩大开放，多路径开展创新，不断释放各方面蕴藏的潜能活力。

　　深化各项改革。继续抓好“三去一降一补”，化解过剩产能，淘汰落后产能；构建多主体供给、多渠道保障、租购并举的住房制度，有序推进商品房库存下降；持续扩大直供电、直供气范围，进一步降低企业运营成本；全面落实补短板十大举措。深化“放管服”改革，把该放的放足、该管的管好；以“十大行动”为抓手，扎实开展“营商环境提升年”活动；争取开展市级相对集中行政许可权改革试点，持续深化行政审批制度改革，推行“互联网+政务服务”，大幅压缩投资项目报建审批时限；积极推广“最多跑一次”经验，着力提高各级政府办事效率和服务水平。深入推进投融资改革，整合县区优质资源资产，将市投控集团资产扩大到200亿元并争取通过外部2A评级，更好发挥投控集团和各类专业基金的撬动作用；全力支持企业在主板、新三板上市，持续扩大民间投资；积极争取各类金融机构在汉设立分支机构，为外埠金融机构来汉开展业务提供方便；支持中小微企业、民营经济和“三农”发展，实现政府融资性担保和农业信贷融资担保机构业务县域全覆盖；严控地方政府债务规模，严厉打击非法集资，有效防范化解金融风险。稳步推进国企、商事、户籍、文化体制等基础性关键性改革，创汉中模式、助汉中发展，为全省创出经验。

　　全面扩大开放。加快建设“四环红太阳”公路网，拓展汉中机场航线，打通区域一体化的交通瓶颈；推进汉中海关和电子口岸建设，加快融入关天、成渝、江汉经济圈，主动对接“一带一路”和长江经济带、京津冀经济圈，使汉中成为企业总部设立分支机构、发展分部经济和学术度假、旅居安居的最佳选择。深入推进区域合作，加快西安“汉中飞地”建设，在融合发展上迈出坚实步伐；抓好“央企入汉”“外企入汉”和“汉品出境”，支持装备制造、机械电子、特色农产品等优势产业抱团出海，在区域产业发展价值链、产业链、供应链中实现资源共享、优势互补、互惠共赢。积极实施“外贸孵化器工程”，新发展10户外贸企业，做大茶叶、柑桔、植物提取物等特色产品出口基地和鹏翔、秦川大厨等出口企业，支持企业到中东欧国家开展经贸合作，推动国际航空转包、感应器、精密齿轮等加工贸易，不断提升经济外向度。

　　大力推动创新。提升区域科技创新支撑能力，加快航空智慧新城高科技制造示范基地建设，推进汉中经济开发区壮大航空配套、食品中药、装备制造等产业，支持汉中高新区全力创建国家级高新区。积极构建以企业为主体的科技创新体系，加快市级和高新区科技资源统筹中心建设，做实做好绿色循环暨产业经济研究院，设立1亿元绿色循环发展科技投资基金，在优势领域择优组建产业技术联盟；加大高新技术企业、瞪羚企业等培育力度，重点培育20家高新技术企业、30户瞪羚企业，新建6个技术研发中心。按照挖掘发展需求、采取针对性激励政策和服务方式灵活多样的思路，吸引一批高新科技和高精尖人才，实现产业需求与科技成果有效对接。推行“孵化+创投”模式，打造一批具有国家水平的创业团队和省级创新创业交流平台，力争每县建立一个创新工场或创业孵化基地，支撑大众创业、万众创新。

　　（七）加快生态文明建设，厚植追赶超越建设“三市”的后发优势。牢记“绿水青山就是金山银山”，把生态文明建设贯穿于经济发展全过程，推进人与自然和谐共生，彰显汉中生产、生活、生态“三生融合”的鲜明特征和独特优势。

　　铁腕整治污染。坚持刚性治标、系统治本，启动新一轮“治污降霾·保卫蓝天”行动，打好减煤、控车、抑尘、治源、禁燃、增绿组合拳，持续改善空气质量，全市优良天数不低于320天，中心城区优良天数达到省考要求、继续减少重污染天气。强力推进污水、垃圾处理设施建设，加大汉江、嘉陵江流域环境突出问题专项整治，确保全域水质达到Ⅱ类标准。加强重金属污染、土壤污染等综合治理，强化固体废弃物处置，确保污染“零增量”，不断减少存量。排查整治日常隐患，坚决防范重特大环境污染事件发生。试行生态环境质量损害赔偿制度。建立“职责法定、岗位明确、层级清晰”的全行业、全层级责任体系，全面完成中省环保督察反馈问题整改任务，启动市级环保督察。

　　系统治理生态。实行最严格的生态环保制度，全面落实河长制和“水十条” “土十条”，扎实开展秦岭破坏生态环境问题“清零”行动和汉江生态环境保护“清澈”行动，年底前全面建立湖长制，统筹推进山、水、林、田、湖、草系统治理。以汉江生态经济带建设为抓手，积极开展国土增绿行动，持续推进天然林保护、退耕还林、水保治理等工程，抓好水网、路网、工矿区、旅游景点等重点区域绿化，不断提高绿化水平和水源涵养能力。严守耕地红线，继续开展高标准农田建设和“旱改水”，确保耕地总量不减、质量不降。深化“三保三治”行动，加强地质灾害监测防治，抓好开山采石专项整治；设立生态修复公司，承担矿山地质环境修复治理和移民搬迁配套工作。探索环境监管和行政执法新路径，构建政府、企业、社会共同参与的环境治理体系。

　　推进绿色循环。围绕将汉江打造成为陕西最美河流，编制《汉江（平川段）生态经济带建设规划》，打造百公里生态廊道和“一个核心区三个增长极”的城镇空间格局，到2025年建成生产总值超过2000亿元的汉江生态经济带。落实主体功能区定位，严格实施重点生态功能区产业准入负面清单，严守资源消耗上线、环境质量底线、生态保护红线。紧盯“双控”目标，大力发展节能环保、清洁能源等绿色产业。实施全民节能行动计划，倡导简约适度、绿色低碳的生活方式，持续扩大绿色消费。

　　（八）全力以赴改善民生，共享追赶超越建设“三市”的发展成果。坚持以人民为中心发展思想，扎实做好保障和改善民生工作。

　　健全完善社保体系。实施更加积极的就业政策，统筹抓好高校毕业生、农民工、就业困难人员、去产能人员等重点群体就业，新增城镇就业3.2万人，转移农村劳动力75万人。全面落实孤儿、高龄老人、残疾人优待政策，切实保障城乡低保、特困群众基本生活；加大社会救助力度，完善农村“三留守”人员关爱服务体系；积极应对人口老龄化挑战，加快老龄事业发展和福利院、敬老院、养老机构建设。扎实做好国防动员、双拥共建等工作，认真落实优抚安置政策和保障待遇，切实维护退役军人合法权益。

　　优先发展教育事业。巩固“双高双普”成果，启动国家义务教育优质均衡区县创建工作，实施教育质量督导评估第三轮“316”工程和第三期学前教育行动计划，推动城乡义务教育一体化发展，着力解决“大班额”、“择校热”、中小学生课外负担重等突出问题。创建普惠性幼儿园。加快推进城乡义务教育公办学校标准化、示范化建设，创建3所省级标准化高中和2所示范化高中。打造职业教育特色品牌，规范社会力量办学，推进终身教育体系建设。出台优惠政策，吸引优质教育资源落户汉中，支持、支撑开放发展。

　　加快健康汉中建设。编制《健康汉中规划》。深化医药卫生体制改革，健全完善分级诊疗、全民医保、药品供应等制度，深入实施基层卫生能力提升三年计划，推进中医药事业传承发展，加强全科医生队伍建设，提高公共卫生服务水平。出台激励政策，引进资金资源，创办高水平医疗机构，支持康养产业发展。在景区、城市、农村全面推进“厕所革命”，不断提升群众生活品质。大力发展体育事业和体育产业，广泛开展全民健身，加快十四届全运会汉中场馆建设，建成滨江体育公园和水上运动中心。

　　持续推进文化振兴。抓好文化惠民工程，完善公共文化体系和文化设施，做优公共文化服务，提升共建共享水平。启动汉中广播电视台多功能演播厅及融媒体指挥中心配套工程建设。全面落实文化产业发展政策措施，发挥好10亿元文化旅游产业发展基金作用。深入挖掘历史、人文、生态资源，精心打造一批文艺精品。大力发展民营文化企业，积极培育新兴文化业态，做大做强文化产业和“汉文化”品牌。

　　大力创新社会治理。扎实开展“安全生产监管能力提升年”活动，细化和落实失职追责、尽职免责的安全生产责任，构建安全生产监管“十个体系”和“双重预防”机制；认真开展安全隐患风险排查治理，加大重点领域、场所、时段的安全生产专项整治力度，特别要着力化解尾矿库隐患，坚决遏制重特大事故发生。强化食品药品监管，严防食品药品安全风险。抓好“七五”普法提档升级载体项目建设。健全“四社联动”机制，发挥群团作用，提升社区治理水平。推进信访法治化建设，加大矛盾纠纷排查化解力度，有效控制进京非访、赴省集体访，抓好信访积案化解和重要节点时期的信访工作。深化平安汉中建设，完善治安防控体系，加强公共安全监管，扎实开展扫黑除恶专项斗争，全力维护和谐稳定。

　　四、建设人民满意的服务型政府

　　建设人民满意的服务型政府，是全市人民的殷切期待。我们必须顺应时代要求，以“不忘初心、牢记使命”主题教育为契机，全面加强自身建设，着力强化“四个意识”、坚定“四个自信”、增进“八大本领”、做到“五个过硬”，为汉中新时代追赶超越建设“三市”提供有力保证。

　　（一）提高政治站位，以新思想引领新时代。服务型政府的核心是忠诚。我们将始终以习近平新时代中国特色社会主义思想为指引，牢牢把握新时代、新思想、新方略的核心要义，引领汉中新时代发展的具体实践；始终站稳政治立场、坚定政治方向、严守政治原则，坚决维护以习近平同志为核心的党中央权威和集中统一领导，永葆对党忠诚、为民造福的政治本色；始终保持政治定力、战略定力、发展定力，不折不扣地贯彻执行中省决策部署，确保政令畅通、令行禁止。

　　（二）始终为民务实，以新举措推动新发展。服务型政府的关键是务实。我们将牢记“人民对美好生活的向往就是我们的奋斗目标”，紧紧抓住群众最关心最直接最现实的利益问题，不断完善公共服务、促进公平正义，使全市人民的获得感、幸福感、安全感更加充实、更有保障、更可持续；牢记“幸福都是奋斗出来的”，勇于挑最重的担子、敢于啃最硬的骨头、善于接最烫的山芋，脚踏实地，苦干实干；牢记“时代是出卷人，我们是答卷人，人民是阅卷人”，多做打基础、利长远的工作，多干解难题、补短板的实事，加快破解不平衡不充分发展问题，努力创造人民认可、经得起历史检验的发展实绩。

　　（三）深化依法行政，以新作为担当新使命。服务型政府的遵循是法治。我们将深入贯彻全面依法治国要求，依照宪法法律行使职权、履行职责；自觉接受人大依法监督和政协民主监督，认真办理人大代表建议和政协提案，坚持邀请人大代表、政协委员列席政府重要会议，严格执行重大行政决策法定程序；深化行政执法体制改革，坚持严格规范公正文明执法，对食品安全、环境污染等重点领域加大执法力度，有效保障群众切身利益；强化行政权力的制约监督，规范土地、矿业权等公共资源管理，深化政务公开，扩大和保障群众的知情权、参与权、表达权、监督权。

　　（四）严守纪律规矩，以新形象凝聚新合力。服务型政府的前提是清廉。我们将坚决落实全面从严治党要求，严守政治纪律和政治规矩，认真履行“一岗双责”主体责任，任何情况下不破底线、不越红线；严格落实中央八项规定实施细则精神和省委实施办法、市委实施意见，深化“激情、干净、超越”主题作风建设，严守优化营商环境规范公职人员行为“十条禁令”，严厉整肃懒政庸政怠政行为，坚决防止“四风”反弹、变异；健全厉行节约反对浪费长效机制，推进财政预决算公开，加强审计监督，不断提高政府工作人员严守纪律、接受监督的自觉，确保“三公”经费只减不增；持续加大反腐力度，坚持无禁区、全覆盖、零容忍，坚持重遏制、强高压、长震慑，严厉整治发生在群众身边的腐败问题，突出抓好扶贫领域腐败和作风问题专项治理，凝聚干事创业、追赶超越的强大合力。

　　各位代表、同志们，新时代的号角高亢而嘹亮，新征程的使命光荣而神圣。让我们以习近平新时代中国特色社会主义思想为指引，在市委的坚强领导下，坚定信心、保持定力，砥砺奋进、真抓实干，在汉中新时代追赶超越建设“三市”的历史进程中谱写新篇章、铸就新辉煌！
